# Efficient PFAS prioritization in non-target HRMS data: systematic evaluation of the novel MD/C-m/C approach

**DOI:** 10.1007/s00216-023-04601-1

**Published:** 2023-02-24

**Authors:** Jonathan Zweigle, Boris Bugsel, Christian Zwiener

**Affiliations:** Environmental Analytical Chemistry, Department of Geosciences, University of Tübingen, Schnarrenbergstraße 94-96, 72076 Tübingen, Germany

**Keywords:** PFAS, High-resolution mass spectrometry, Non-target-screening, Feature prioritization, Data reduction, Elemental composition

## Abstract

Non-target screening (NTS) based on high-resolution mass spectrometry (HRMS) is necessary to comprehensively characterize per- and polyfluoroalkyl substances (PFAS) in environmental, biological, and technical samples due to the very limited availability of authentic PFAS reference standards. Since in trace analysis, MS/MS information is not always achievable and only selected PFAS are present in homologous series, further techniques to prioritize measured HRMS data (features) according to their likelihood of being PFAS are highly desired due to the importance of efficient data reduction during NTS. Kaufmann et al. (*J AOAC Int*, 2022) presented a very promising approach to separate selected PFAS from sample matrix features by plotting the mass defect (MD) normalized to the number of carbons (MD/C) vs. mass normalized to the number of C (m/C). We systematically evaluated the advantages and limitations of this approach by using ~ 490,000 chemical formulas of organic chemicals (~ 210,000 PFAS, ~ 160,000 organic contaminants, and 125,000 natural organic matter compounds) and calculating how efficiently, and especially which, PFAS can be prioritized. While PFAS with high fluorine content (approximately: F/C > 0.8, H/F < 0.8, mass percent of fluorine > 55%) can be separated well, partially fluorinated PFAS with a high hydrogen content are more difficult to prioritize, which we discuss for selected PFAS. In the MD/C-m/C approach, even compounds with highly positive MDs above 0.5 Da and hence incorrectly assigned to negative MDs can still be separated from true negative mass defect features by the normalized mass (m/C). Furthermore, based on the position in the MD/C-m/C plot, we propose the estimation of the fluorine fraction in molecules for selected PFAS classes. The promising MD/C-m/C approach can be widely used in PFAS research and routine analysis. The concept is also applicable to other compound classes like iodinated compounds.

**Graphical Abstract:**

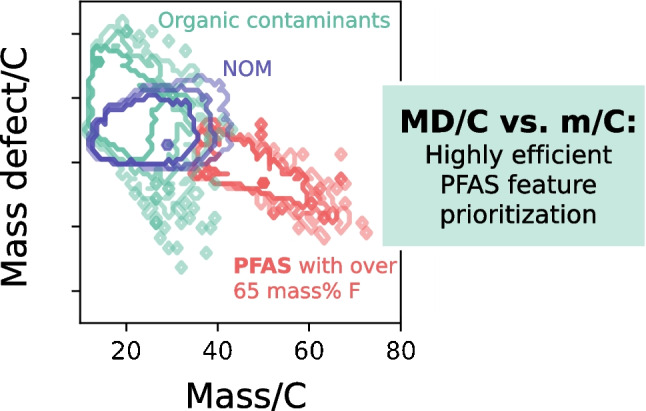

**Supplementary Information:**

The online version contains supplementary material available at 10.1007/s00216-023-04601-1.

## Introduction  

Per- and polyfluoroalkyl substances (PFAS) are an immense class of anthropogenic chemicals with useful properties for countless commercial applications [1, 2]. PFAS characterized by C_n_F_2n+1_– or C_n_F_2n+1_–O–C_m_F_2m_–units exhibit non-stick properties and extreme stability [3, 4]. This high persistence, of either PFAS themselves or their perfluorinated transformation products, led to a global distribution of perfluoroalkyl acids (PFAAs) [5, 6]. As a reason of this property, combined with their bioaccumulation potential and observed adverse health effects, much effort was put into regulations for selected long-chain PFAS (perfluoroalkyl carboxylic and sulfonic acids) [7]. However, the development of replacement compounds and the large market for PFAS continuously increases the number of individual fluorinated substances and their production volume [8]. Several studies showed considerable fractions of unidentified organic fluorine in numerous samples, including human serum, showing that much more unknown PFAS must be present in the environment [9–12].

Since the sheer number of PFAS makes a comprehensive use of authentic analytical reference standards practically impossible, non-target screening (NTS) approaches based on high-resolution mass spectrometry (HRMS) are a necessary tool for PFAS identification in all kinds of samples [13, 14].

In NTS, data reduction and prioritization of features is always a crucial step for an efficient workflow. The chemical mass defect (MD), which is typically slightly negative for PFAS with high fluorine content, can be used as a first prioritization approach [15, 16]. For PFAS that occur as homologous series in samples of interest, Kendrick mass defect (KMD) analysis is a powerful tool for data prioritization and compound identification [17, 18]. In case of relatively high PFAS concentrations compared to the sample matrix, approaches relying on MS^2^ data using diagnostic fragments and/or fragment mass differences are efficient in detecting potential PFAS candidates and further identifying them [19, 20]. However, approaches that rely on MS^2^ data for prioritization are often impractical in trace analysis, because achieving a broad MS/MS coverage can be very time consuming and exhaustive coverage is usually not possible due to detection limits and hence noisy mass spectra. Furthermore, unknown PFAS not occurring as homologues cannot be captured by KMD analysis and if present in trace concentrations further homologues might not be present in sufficient concentrations for the peak picking algorithm or lost during certain data reduction steps. Depending on the sample matrix, even the MD approach may fail for compounds with a high positive MD that exceeds + 0.5 Da that can be erroneously interpreted as negative MD and therefore incorrectly assigned to PFAS (see Fig. S1). Data filtering with too strict MD ranges on the other hand may exclude true positives (mainly critical for PFAS with high H-content or other halogens).

**Fig. 1:**
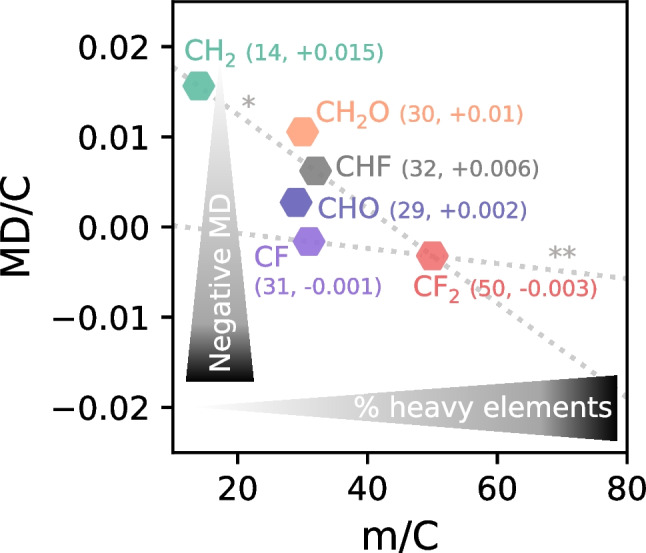
Schematic explanation of the MD/C-m/C plot. Important positions of compounds composed on average of CH_2_, CH_2_O, CHO, CHF, CF, and CF_2_ are shown. The general trend of increasing m/C with increasing percentage of heavy elements (e.g., halogens, O, N, P, S, and heavy metals) and the decreasing trend of MD/C with increasing numbers of elements that have a negative mass defect are highlighted. Furthermore, the CH_x_F_2-x_-line (*, with 0 ≤ x ≤ 2, see Eq. 1) and the CF_x_-line (**, with 0 ≤ x ≤ 2, see Eq. 2) are given as orientations (see Fig. S2 for discrete points that fall on those lines)

In a recent publication, Kaufmann et al. (2022) presented a highly promising approach for an efficient prioritization of potential PFAS in complex matrices (fish extracts) [21]. Compounds with high fluorine content (composed mainly of C and F) have much lower carbon numbers compared to compounds dominated by C and H at a similar mass. The carbon number can be retrieved from HRMS data from the abundance of the ^13^C isotope [M + 1] according to the following equation: *C* = *I*_M+1_/*I*_M_/0.011145, where *I*_M+1_ and *I*_M_ correspond to the intensities of the first isotopic and monoisotopic peak, respectively [21]. Therefore, a compound mass normalized to the number of C atoms (m/C) can be used as a separation criterion for potential PFAS features (for CF_2_, m/C = 50) from matrix features (for CH_2_, m/C = 14). A further criterion for PFAS selection is the MD normalized to the number of C atoms (MD/C), for which Kaufmann et al. (2022) observed a strong separation of PFAAs from fish matrix in NTS data due to the typically more negative MD/C of PFAS compared to other CHO compounds. The general concept of the MD/C-m/C plot is illustrated in Fig. 1. Compounds with an increased number of heavier elements compared to H are shifted to higher m/C values, while compounds with a higher number of elements with negative MD are shifted to a more negative MD/C. As an example, a PFAS for which the chemical formula approaches (CF_2_)_n_ would plot at m/C ≈ 50 and MD/C ≈ − 0.003 while a compound mainly composed of (CH_2_O)_n_ would plot at m/C = 30 and MD/C =  + 0.0106, showing that such a separation generally works. Therefore, if we consider compounds which are mainly characterized by the transition from a saturated hydrocarbon (CH_2_) to a perfluoroalkyl substance (CF_2_) (CH_x_F_2-x_, x = 0, 1, 2) all plot along the following line:

1 $$\mathrm{MD/{C}_{{CH}_{x}{F}_{2-x}}}\approx -5.24\times {10}^{-4}\cdot \mathrm{m/C}+0.023$$

The same principle holds for compounds that are characterized by the transition between CF and CF_2_:

2 $$\mathrm{MD/{C}_{{CF}_{x}}}\approx -8.406\times {10}^{-5}\cdot \mathrm{m/C}+0.001$$

with 0 ≤ x ≤ 2. Both lines can be used as a very helpful orientation when using the MD/-m/C plot for PFAS feature prioritization (discussed later, see also Fig. 1). One further important and useful intrinsic property of the MD/C-m/C plot is that structurally related compounds are clustered together.

To investigate the efficiency of the MD/C-m/C approach and its robustness to prioritize features as potential PFAS in GC or LC-HRMS data, we used chemical formulas of ~ 490,000 organic chemicals from online sources (PubChem (https://pubchem.ncbi.nlm.nih.gov) and Koch et al. 2007) [22, 23]. We systematically evaluated which chemical composition of PFAS classes (~ 210,000 compounds) can be separated from natural organic matter (NOM) compounds (~ 125,000) representing a typical matrix of environmental samples and from other organic contaminants (~ 160,000) and how explicitly that is possible. The degree of fluorination in a PFAS was expressed as F/C ratio (number of fluorine atoms divided by number of carbon atoms), H/F ratio (number of hydrogen atoms divided by number of fluorine atoms), mass percentage of fluorine (%m_F_), and by the molar percentage of F atoms per molecule (%n_F_) to determine the range of elemental compositions (chemical formulas) for a clear separation from matrix components. General advantages and limitations of the MD/C-m/C approach are discussed in detail. Furthermore, we discuss the possibility to estimate the degree of fluorination of a compound based on its position in the MD/C-m/C plot for a statistically relevant number of PFAS.

## Methods

### Data collection

To perform a data evaluation with a robust amount of organic chemicals, raw data was downloaded from the PubChem Classification Browser and from the Supporting Information of Koch et al. 2007 [22–24] and preprocessed in three individual datasets which are PFAS, organic compounds (OCs), and NOM compounds. From PubChem, the EPA DSSTox dataset (245,545 compounds) [25], the NORMAN Suspect List Exchange (113,737 compounds) [26] and from the “PFAS and Fluorinated Compounds in PubChem Tree” PFAS with parts larger than CF_2_ or CF_3_ that fall into the OECD definition (224,017 compounds) were downloaded as CSV and TXT files [27, 28]. The EPA DSSTox dataset includes any kind of toxic substances while the NORMAN database includes emerging environmental contaminants. To also include natural substances (NOM) which are typical matrix compounds in LC–MS (and GC) measurements, chemical formulas of 124,782 NOM constituents derived from ESI FT-ICR-MS measurements of a Suwannee River Fulvic Acid Standard (SRFA II) were included [22].

### Data cleanup

Data cleanup and calculations were performed with Python 3.9.13. Each CSV file was imported, and several cleanup steps and basic chemical calculations were performed which are presented in the following bullet points:

•Masses below 100 Da and above 2000 Da were removed to obtain a reasonable GC- and LC-MS mass range.
•All inorganic compounds were removed (mainly present in the EPA DSSTox dataset).
•All salts were removed via periods in their SMILES code.
•All organometallic compounds were removed since they usually play a minor role in typical environmental matrices and in addition most of them can rather easily be distinguished from other organic molecules by their unique isotopic pattern. Approximately 5% organometallic compounds were present in the three PubChem databases. Only compounds containing C, H, N, O, P, S, Si, F, Cl, Br, and I were kept for further calculations.
•The exact mass was calculated for each compound from its molecular formula. In case of charged compounds (e.g., quaternary ammonium compounds), charges were removed for the mass calculation.
•The number of C, F, and H atoms and the total number of atoms per compound were calculated.
•Both exact MD (theoretical; sum of MDs of all elements in the chemical formula) and the calculable MD (MD = accurate mass − integer mass) were determined.
•Finally, m/C and MD/C were calculated for all compounds. Additionally, the MD/C was calculated with the calculable MD.

In the following step, the EPA DSSTox and NORMAN database were combined and most overlapping compounds (the EPA DSSTox already contains part of NORMAN) were removed by keeping only unique SMILES and InChIKeys. Then all fluorine-containing compounds were removed. This final dataset contained 182,503 organic contaminants without fluorine which are denoted as OCs in the following. The NOM dataset was kept in its original form with 124,782 compounds containing C, H, O, N, S, and P.

For the PFAS dataset (210,091 PFAS), four parameters were calculated that describe the amount of fluorine in PFAS molecules in different ways: F/C ratio, H/F ratio, the mass percentage of fluorine (%m_F_), and the fraction of fluorine atoms per molecule (%n_F_).

### Data evaluation

#### Separation of PFAS from OCs and NOM

To determine how well PFAS can be separated from compounds without fluorine (OCs and NOM) in the MD/C-m/C plot a MD/C vs. m/C domain from − 0.05 ≤ MD/C ≤ 0.025 and 10 ≤ m/C ≤ 100 and was chosen which included > 99.8% of all compounds of the three groups. Compounds that fall out of this domain were not included in the data evaluation (mainly compounds with less than two C atoms and heavy elements such as I or Cl and P).

To determine the position of each class in the MD/C-m/C domain, this was subdivided in a rectangular grid of 2D bins. A grid size of either 70 × 70 or 100 × 100 bins was chosen which corresponds to bin sizes of 0.0011 MD/C × 1.28 m/C or 0.00075 MD/C × 0.9 m/C. For the binned data, a 2D histogram was calculated for all three compound groups. The resulting matrix with the number of compounds in each bin (counts) was normalized to the total number of compounds present to obtain the fraction of compounds relative to all compounds in each bin (the sum of the normalized matrix corresponds to 100%) (Fig. S3). To find the position of a certain percentage of compounds (e.g., 90%) around the region with highest density of compounds, the bins were summed up in decreasing order until the desired percentage criterion was reached (a schematic explanation of this procedure is depicted in Fig. S3). Now the matrix of PFAS can be compared to the matrix of the other compound classes (OCs or NOM) to find the overlapping region of both classes and determine the fraction of compounds that overlap for both classes (Fig. S4). Since OCs with high amounts of heavy elements (e.g., Br or I) always overlap with some PFAS, the calculations were performed for different percentages of each class. With this general procedure, the overlap was simulated for different fluorine content by varying the parameters F/C, H/F, %m_F_, and %n_F_ and considering PFAS that fall into the criterion (e.g., PFAS with %m_F_ ≥ 50%).

#### Position of PFAS in the MD/C-m/C plot as a function of fluorine content

To determine the distribution of F/C, H/F, %m_F_, and %n_F_ in the MD/C-m/C plot for PFAS, the mean and standard deviation of PFAS with %m_F_ > 50% in each bin of the 2D histogram were calculated (see Fig. S3b for a schematic explanation). The 2D matrices with the mean of F/C, H/F, %m_F_, and %n_F_ in each bin were used to investigate how well single bins represent these parameters (F/C, H/C etc.) and therefore how well MD/C-m/C positions can be used to predict those (F/C, H/C etc.) for PFAS. Furthermore, the overall error distribution was determined from the standard deviation matrices to conclude on the precision of such a prediction.

## Results and discussion

### Separation of PFAS from NOM and organic contaminants (OCs)

**Fig. 2:**
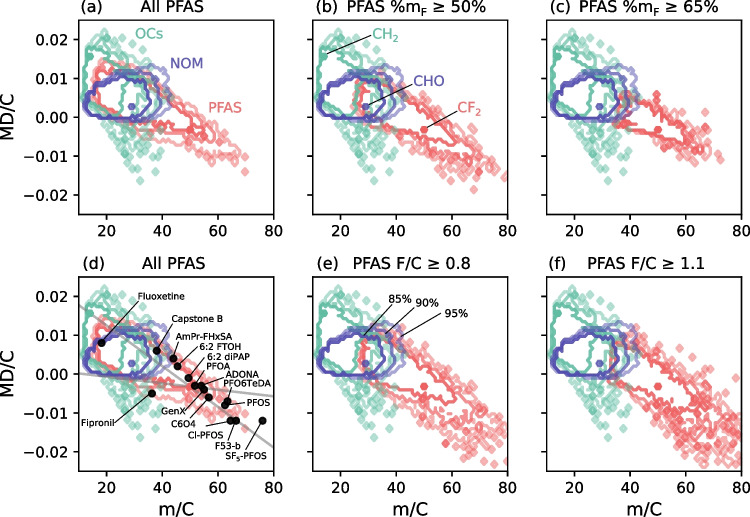
Positions of organic contaminants (OCs, green) from the EPA DSSTox and NORMAN (182,503), NOM (blue) (124,782), and PFAS (red) (all: 209,760) with different amounts of fluorine (according to mass percent F (%m_F_) (**a**–**c**) and F/C ratio (**d**–**f**)) in the MD/C-m/C plot. The contour lines delimit the positions of 80% (center), 90% (middle), and 95% (external) of each group. In case of PFAS with %m_F_ ≥ 65% or F/C ≥ 1.1, an almost complete separation is possible. Details on the known PFAS shown in subplot (**d**) are given in Table 1. The CH_x_F_2-x_-line (with 0 ≤ x ≤ 2) and the CF_x_-line (with 0 ≤ x ≤ 2) are given as orientations (see also Fig. 1; for discrete data points of PFAS that fall on the CH_x_F_2-x_-line and CF_x_-line, see Fig. S2). Figure S6 provides another representation of the overlap as further information

The position of organic compounds in the MD/C-m/C plot depends on their average mass per C atom and their average MD per C atom which are both strongly correlated with chemical composition. Molecules with multiple heavy elements (e.g., halogens, O, S, P, heavy metals) rather than H are shifted to the lower right corner (lower MD/C and higher m/C). This can be used to separate features that are highly fluorinated from other organic contaminants (OCs) and NOM (Fig. 2). The positions for 80%, 90%, and 95% of OCs (182,503 compounds from the EPA DSSTox and NORMAN database), typical NOM constituents (124,782), and PFAS (209,760 with more than one CF_2_ or CF_3_ group according to the OECD) were calculated and visualized in the MD/C-m/C plot. Their exact distribution in the MD/C-m/C domain is shown in 2D histograms (Fig. S5).

**Table 1:** m/C, MD/C, H/F, F/C, %m_F_, and %n_F_ for well-known PFAS which are shown in Fig. 1. Abbreviations: *SF*_*5*_*-PFOS*, pentafluorosulfanyl perfluorooctane sulfonic acid [29]; *F53-b*, 6:2 Cl-perfluoro ether sulfonic acid; *PFO6TeDA*, perfluoro- (3,5,7,9,11,13-hexaoxatetradecanoic) acid [30]; *PFOS*, perfluorooctane sulfonic acid; *C6O4*, perfluoro ([5-methoxy-1,3-dioxolan-4-yl]oxy) acetic acid; *GenX or HFPO-DA*, hexafluoropropylene oxide dimer acid; *ADONA*, dodecafluoro-3H-4,8-dioxanonanoate; *PFOA*, perfluorooctanoic acid; *6:2 diPAP*, 6:2 polyfluoroalkyl phosphate diester; *6:2 FTOH*, 6:2 fluorotelomer alcohol; *AmPr-FHxSA*, N-dimethyl ammonio propyl perfluorohexane sulfonamide [31]; *Capstone B* (also 6:2 FTAB), 6:2 fluorotelomer sulfonamide alkylbetaine [32]. For the predicted F/C, the table provided as Electronic Supplementary Material was used. Compounds without prediction fell either in bins outside the used MD/C-m/C domain or were located in bins with less than 5 entries which were excluded in data analysis.

Compound	Formula	m/C	MD/C	H/F	F/C	%m_F_	%n_F_	Predicted F/C
SF_5_-PFOS	C_8_F_21_O_3_S_2_	76.0	− 0.012	0.05	2.63	66	60	-
F53-b	C_8_HClF_16_O_4_S	66.5	− 0.012	0.06	2.00	57	52	2.0
Cl-PFOS	C_8_HF_16_ClO_3_S	64.5	− 0.012	0.06	2.00	59	53	-
PFO6TeDA	C_7_HF_13_O_7_	63.4	− 0.007	0.08	1.86	56	46	2.2
PFOS	C_8_HF_17_O_3_S	62.5	− 0.008	0.06	2.13	65	57	2.2
C6O4	C_6_HF_9_O_6_	56.7	− 0.006	0.11	1.50	50	41	1.9
GenX	C_6_HF_11_O_3_	55.0	− 0.004	0.09	1.83	63	52	1.9
ADONA	C_7_H_2_F_12_O_4_	54.0	− 0.003	0.17	1.71	60	48	1.9
PFOA	C_8_HF_15_O_2_	51.7	− 0.003	0.07	1.88	69	58	1.9
6:2 diPAP	C_16_H_9_F_26_O_4_P	49.4	− 0.001	0.35	1.63	63	46	1.8
6:2 FTOH	C_8_H_5_F_13_O	45.5	+ 0.002	0.38	1.63	68	48	1.5
AmPr-FHxSA	C_11_H_13_F_13_N_2_O_2_S	44.0	+ 0.004	1.00	1.18	51	32	1.4
Capstone B	C_15_H_19_F_13_N_2_O_4_S	38.0	+ 0.006	1.46	0.87	43	24	1.2
Fipronil	C_12_H_4_Cl_2_F_6_N_4_OS	36.3	− 0.005	0.67	0.50	26	20	-
Fluoxetine	C_17_H_18_F_3_NO	18.2	+ 0.008	6.00	0.18	18	8	-

Overall, many partly fluorinated PFAS which are dominated by CH from the PubChem dataset overlap with both OCs and NOM while highly fluorinated PFAS are well separable if they are characterized by %m_F_ >  ~ 65% or F/C ratios ≥  ~ 1.1 (Fig. 2). High fluorine content draws the compounds the lower right corner of the MD/C-m/C plot. In Fig. S6, another representation of the overlap is provided as additional information.

**Fig. 3:**
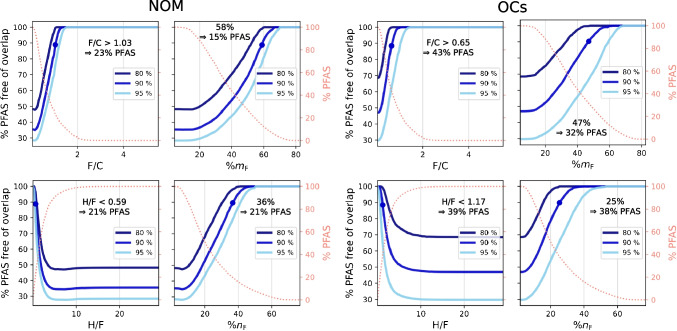
Simulation of the separation of PFAS dependent on their fluorine content (> F/C, < H/F, > %m_F_, and > %n_F_) from NOM compounds and organic contaminants (OCs) in the MD/C-m/C plot for 80%, 90%, and 95% of both classes (NOM vs. PFAS and OCs vs. PFAS). Blue dots mark the position where 90% of PFAS overlap less than 10% with 90% of the other class. The secondary *y*-axis shows the PFAS fraction that is above (F/C, %m_F_, %n_F_) or below (H/F) each fluorine quantity parameter. The text within each subplot gives the exact fluorine quantity and how much PFAS fall into that range (PFAS that have > F/C, %m_F_, and %n_F_ and for H/F PFAS that have < H/F) for 90% of both classes. The underlying concept of the calculation of one data point is exemplified in Fig. S4

**Fig. 4:**
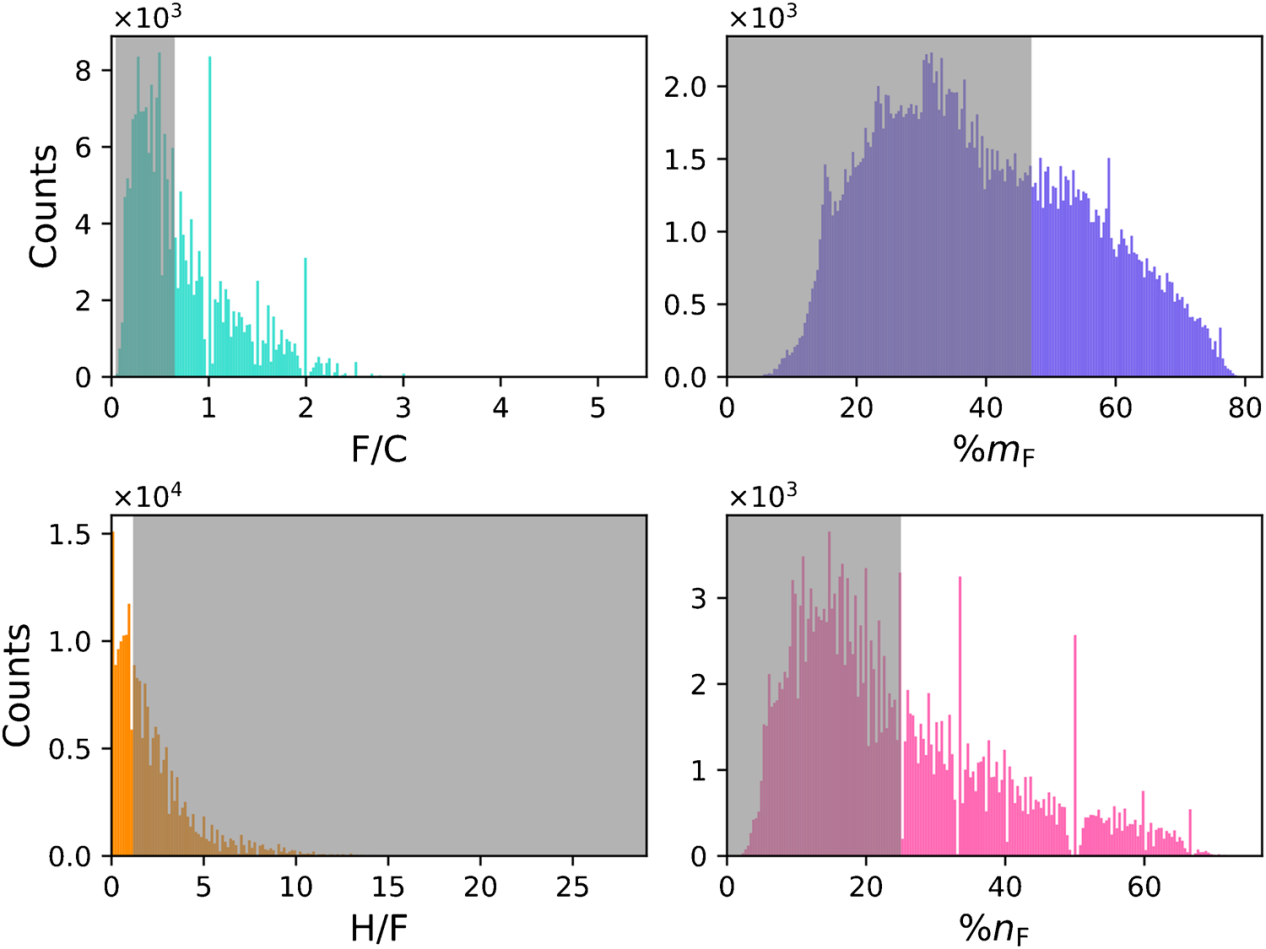
Histograms of the distributions of PFAS with larger parts than CF_2_ or CF_3_ (210,091 compounds) from PubChem for F/C, H/F, %m_F_, and %n_F_. Grey areas indicate PFAS that cannot be easily separated by the MD/C-m/C approach based on the 90%/90% boundaries from Fig. 3 since they overlap with other compounds. The grey areas are given according to the calculations based on OCs. The data for each histogram was separated in 200 bins. Note which molecular compositions of PFAS are frequent. Note that the PFAS inside the grey area are not necessarily “non-separable PFAS”

To put the overlap into a quantitative context, the separation of PFAS from both NOM and OCs was simulated individually by varying F/C, H/F, %m_F_, and %n_F_ (Fig. 3). In general, NOM compounds show more overlap with PFAS in the critical lower MD/C and higher m/C range. From these calculations, approximate boundaries for an efficient PFAS separation can be estimated. In the case of NOM, 90% of the PFAS are separated from 90% of the NOM constituents (or in other words less than 10% of PFAS are in overlapping regions with NOM features), if the F/C ratio of PFAS is higher than ~ 1.03, or the H/F ratio smaller than ~ 0.59, or the %m_F_ higher than ~ 58% or the fraction of fluorine atoms in the chemical formula is at least ~ 36% (for details, see Fig. 3). For the OCs, the boundaries (90%/90%) are F/C ~ 0.65, H/F ~ 1.17, %m_F_ ~ 47%, or %n_F_ ~ 25%. Histograms for PFAS are given in Fig. 4, where non-separable PFAS that fall below the thresholds of F/C, %m_F_, and %n_F_ or above H/F are covered by a grey area (90%/90% boundary). It is important to note that the determined boundaries should be considered as smooth transitions, as they are shown in Fig. 3 and the PFAS in the grey area are not necessarily “non-separable”.

In principle, these calculations reflect a worst-case scenario because in typical HRMS measurements, only a much smaller number of features is present (especially because a sufficient ^13^C isotope signal is required which further reduces the feature number) that directly compete with potential PFAS in the MD/C-m/C plot. However, the potential overlap in real-world measurements is always highly dependent on the sample matrix. In the case of fish extracts measured by Kaufmann et al. (2022), a complete separation of PFAS from co-extracted matrix from liver and muscle tissue was achieved. We further included four MD/C-m/C plots of PFAS in extracts of agricultural soils (raw data from Bugsel and Zwiener 2020 [17]) to demonstrate the applicability of the approach in the SI (details in Fig. S7).

In the case of the presence of compounds with low MD/C and high m/C (e.g., halogenated substances, organometallic compounds, and others), a distinct separation from PFAS might not always be possible. Nonetheless, such compounds can often be separated from PFAS by their distinct isotopic patterns (e.g., Cl, Br, many heavy metals). This is more difficult for compounds with I or high percentages of O, P, or S. The MD/C-m/C plot is therefore also highly useful to find other compound classes such as iodinated substances, since they are located at even higher m/C and lower MD/C values than PFAS. The MD/C-m/C approach for PFAS feature prioritization has a further key advantage over the use of the MD alone. Compounds with a high positive MD above + 0.5 Da (for the simplest case (CH_2_)_32_ = 448.5008 Da, becomes more important with increasing mass) may erroneously interpreted as negative MD (rounding to the nearest integer) and hence incorrectly assigned to highly halogenated compounds like PFAS (see Fig. S1). In particular, this becomes more important for samples with high-molecular weight PFAS. In the MD/C-m/C plot, this unavoidable issue becomes much less of a problem because such features (high H content and MD >  + 0.5 Da) are still separated by the m/C dimension. For example, m/C of (CH_2_)_32_ = 14 compared to m/C of CHF (= 32) or CF_2_ (= 50) (see Fig. S8).

To classify the positions of selected examples of known PFAS in the MD/C-m/C plot, they were included in Fig. 2d (abbreviations are given in the caption of Table 1). For high fluorine-containing PFAS, a separation is clearly possible with limitations in case of the telomer-based aqueous film-forming foam (AFFF) compound Capstone B and the pharmaceutical fluoxetine. The separation works also very well for ether-PFAS such as C6O4, ADONA, GenX, and PFO6TeDA due to an additional high O content. AmPr-FHxSA (electrochemically fluorinated AFFF), 6:2 FTOH, and fipronil are examples (low MD/C due to 2 Cl atoms and high m/C resulting from a high fraction of heteroatoms) that fall in regions closer to some OCs and NOM. Fluoxetine has only one CF_3_-group and is an example for a compound that cannot be prioritized due to the dominance of CH in the chemical formula. This is generally the case for all compounds with low CF compared to CH groups which are therefore shifted to the upper left part of the MD/C-m/C-plot and hence overlapping with NOM constituents (CHO) and many other organic chemicals. Information on the F/C, H/F, %m_F_, and %n_F_ of those PFAS examples are given in Table 1. In general, all examples with longer perfluoroalkyl (or perfluoroether) chains are in the vicinity of the CH_x_F_2-x_-line as indicated in Fig. 2d (see also Fig. 1 and Eq. 1). This line can be used as a helpful tool to estimate the elemental composition of features that are located closely and helps as an orientation in the MD/C-m/C plot. Compounds with more H atoms are shifted upwards in MD/C (e.g., 6:2 FTOH, or the AFFF compounds) while the presence of 2 Cl atoms in fipronil results in a lower MD/C which shifts downwards (and a shift to the right at higher m/C).

Generally, the prioritization of features as potential PFAS in the MD/C-m/C plot should either be performed elliptically outwards from the CF_2_ position (MD/C ≈ − 0.003 and m/C ≈ 50) along the slope of the CH_x_F_2-x_-line (see Eq. 1) or along the CH_x_F_2-x_-line from right to left (increasing MD/C and decreasing m/C). To facilitate the procedure, we propose to rotate the MD/C vs. m/C data by the angle of the CH_x_F_2-x_-line and to shift the CF_2_ position to the origin (0,0) (Eqs. 3 and 4):

3 $$\mathrm{m/}{\mathrm{C}}_{m}\mathrm{ = }\left(\mathrm{m/C }-{\mathrm{ m/C}}_{{\mathrm{CF}}_{2}}\right){\mathrm{cos}}\left({m}\right) \, - \, \left(\mathrm{MD/C }- \, {\mathrm{MD/C}}_{{\mathrm{CF}}_{2}}\right)\mathrm{sin(}{m}\mathrm{)}$$

4 $$\mathrm{MD/}{\mathrm{C}}_{m}\mathrm{ = }\left(\mathrm{m/C }- \, {\mathrm{m/C}}_{{\mathrm{CF}}_{2}}\right){\mathrm{sin}}\left({m}\right)\mathrm{ + }\left(\mathrm{MD/C }- {\mathrm{MD/C}}_{{\mathrm{CF}}_{2}}\right)\mathrm{cos(}{m}\mathrm{)}$$

where m/C_*m*_ and MD/C_*m*_ are the new shifted and rotated locations, ${\mathrm{m/C}}_{{\mathrm{CF}}_{2}}$ and ${\mathrm{MD/C}}_{{\mathrm{CF}}_{2}}$ are the respective CF_2_ positions (49.9968, − 0.00319), and *m* is the positive slope of the CH_x_F_2-x_-line (+ 5.23 × 10^−4^). For prioritization of subsets of the features, suitable ranges for MD/C_*m*_ and MD/C_*m*_ can now be set more easily (e.g., ± 10 m/C and ± 0.001 MD/C) (see Fig. S9). Furthermore, if a continuous feature prioritization is desired (ranking), the elliptical radii of features from the shifted CF_2_ position (0,0) can be calculated according to Eq. 5:

5 $${\mathrm{r}}_{{\mathrm{CF}}_{2}}\mathrm{ = }\sqrt{{\left(\frac{\mathrm{m/}{\mathrm{C}}_{m}}{\lambda }\right)}^{2}+ \mathrm{ } {\mathrm{MD/}{\mathrm{C}}_{m}}^{2}}$$

where ${\mathrm{r}}_{{\mathrm{CF}}_{2}}$ corresponds to the radial distance from the CF_2_ position and *λ* is a factor that determines the aspect ratio of the ellipse. Since the m/C range of PFAS with high F content (e.g., %m_F_ > 60%) is approximately 3000 times the MD/C range (see Figs. S9 and S10), a reasonable *λ* would be ~ 3000; however, this parameter should be adjusted. After calculating ${\mathrm{r}}_{{\mathrm{CF}}_{2}}$, features can be prioritized by sorting them by increasing ${\mathrm{r}}_{{\mathrm{CF}}_{2}}$. It should be noted that *λ* is an empirical parameter that should be interpreted as an approximate value.

### Estimation of the degree of fluorination

**Fig. 5:**
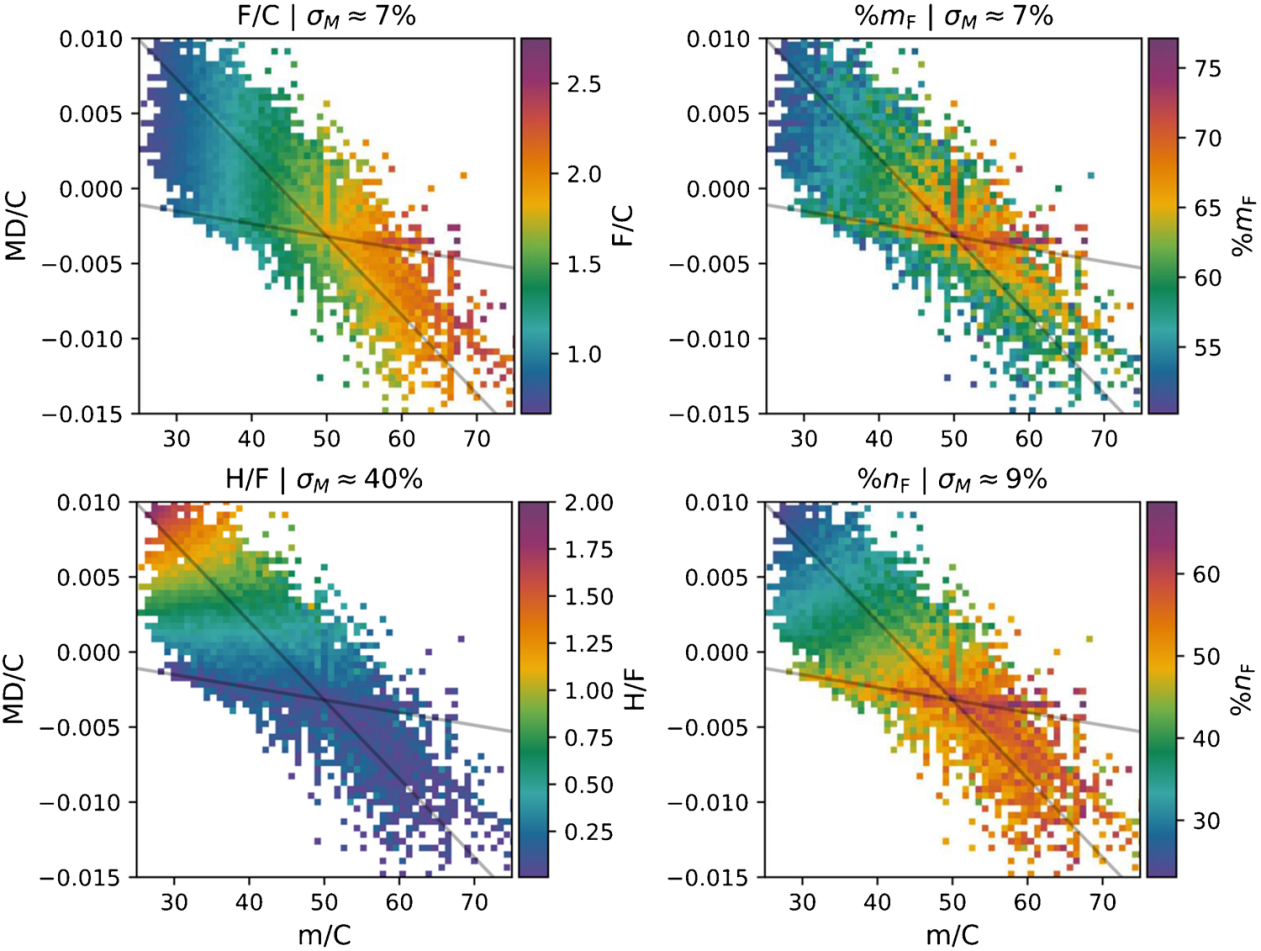
Average F/C, H/F, %m_F_, and %n_F_ for PFAS with %m_F_ > 50% in the MD/C-m/C plot (70 × 70 bins). The F/C ratio can be roughly estimated from a feature position in the MD/C-m/C plot (for correlation, see Fig. 6). Plot titles (σ_*M*_) provide the median overall standard error (for standard error distribution, see Fig. 7). Exact standard deviations in each bin are provided in Fig. S12. Due to the higher variability of H/F, %m_F_, and %n_F_ independent on the position in the MD/C-m/C plot, no accurate predictions can be performed (see Fig. S13 for correlation plots)

To determine whether the degree of fluorination can be estimated from the position of a PFAS feature in the MD/C-m/C plot, the mean and standard deviations of PFAS with %m_F_ > 50% in each MD/C-m/C bin (70 × 70 grid) were calculated for F/C, H/F, %m_F_, and %n_F_ (Fig. 5). Only PFAS with more than 50% m_F_ were used in this data analysis because 50% m_F_ was approximately determined to be sufficient for a separation of PFAS from other compounds (see Fig. 3). Bins in which less than 5 PFAS are located were excluded from the analysis since 5 compounds have been considered as minimum for statistical calculations like mean and standard deviation. It is important to mention that the standard deviation in each bin is dependent on the grid size, since a very fine grid (only one compound per bin) would result in a variance of zero. We have chosen a grid size of 70 × 70 bins for this data evaluation (for the dependency of the standard error on grid size, see Fig. S11; further details on calculations in Fig. S3).

For F/C, H/F, %m_F_, and %n_F_, overall reproducible distributions in the MD/C-m/C plot were observed (Fig. 5). The F/C ratio increases with increasing m/C (from left to right). H/F shows a decreasing trend with decreasing MD/C with particularly small values slightly above and below the CF_x_-line. %m_F_ shows an elliptical decreasing trend (along the CH_x_F_2-x_-line and other specific positions) with increasing distance to the CF_2_-point and maxima along the specific lines (Fig. 5). A similar distribution was observed for %n_F_; however, in the region higher than m/C = 50, it still increases with increasing m/C. For all four fluorine parameters (F/C, H/F, etc.), there are exceptions which originate from the underlying dataset.

**Fig. 6:**
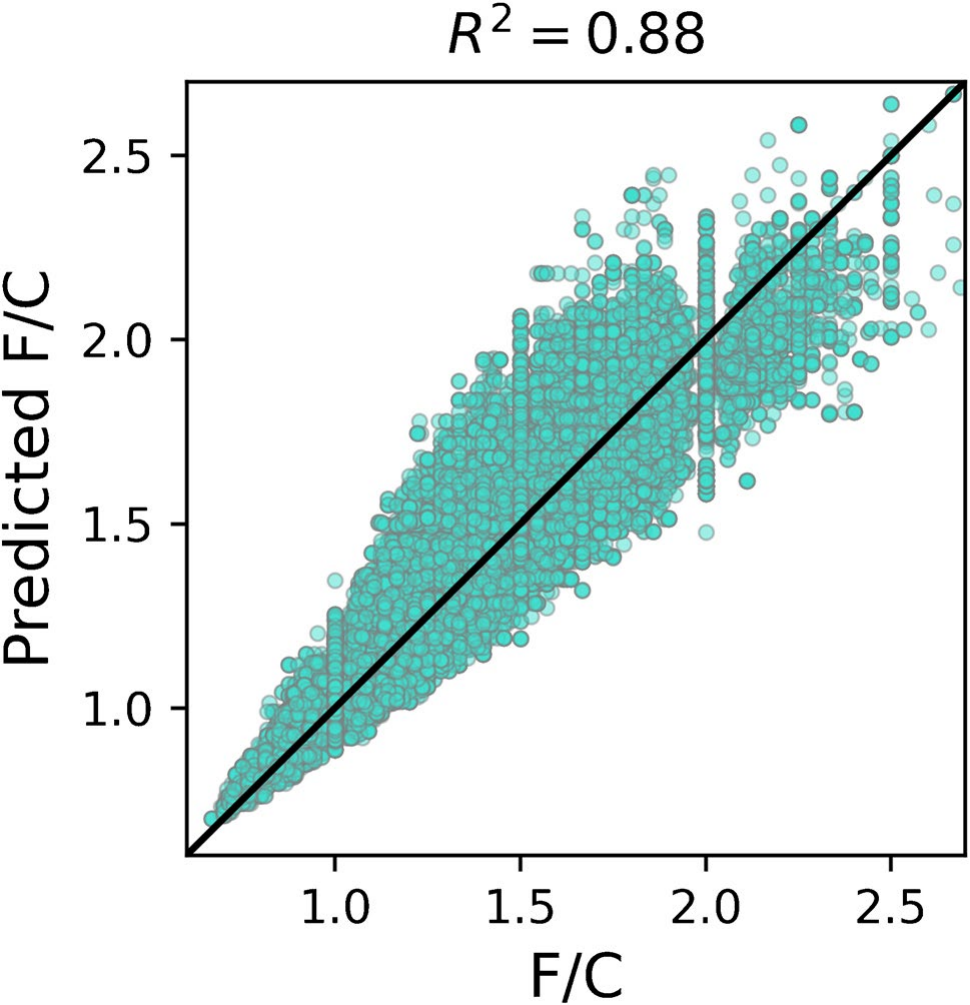
Predicted F/C ratios based on the MD/C-m/C position in the mean matrix of F/C (F/C subplot in Fig. 5 and the Excel table of the ESI) vs. calculated real F/C ratios for chemical formulas of 52,769 PFAS with %m_F_ > 50%. Predictions for H/F, %m_F_, and %n_F_ are given in Fig. S13

**Fig. 7:**
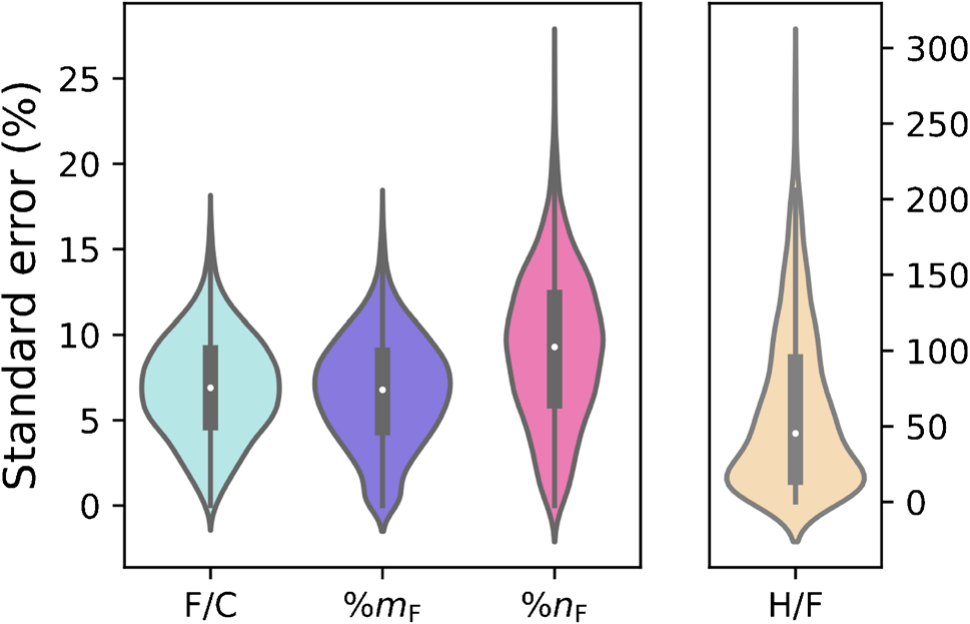
Distribution of the standard error of F/C, %m_F_, %n_F_, and H/F in all bins from the MD/C-m/C plot for the calculations in Fig. 5 (for detailed standard deviations and calculations, see Figs. S3 and S12). While F/C, %m_F_, and %n_F_ have reproducible errors, as H/F approaches close to zero, the standard error becomes very high due to division by a very small mean H/F (see also Fig. S13). Therefore, a reasonable prediction of H/F cannot be achieved from a position in the MD/C-m/C plot

To estimate how well the four fluorine parameters can be estimated from a position in the MD/C-m/C plot, they were predicted based on the respective calculated 2D mean matrices. Those predicted values were then correlated with the true values (see Fig. 6 for F/C, and Fig. S12 for H/C, %m_F_, and %n_F_). Obviously, for F/C and H/F, the strongest correlation (*R*^2^ = 0.88) was observed while for %n_F_ and %m_F_, it was lower (0.72 and 0.41, respectively). The distribution of the standard errors of the mean in all bins reveals that H/F has by far the largest standard error (see Fig. 7; the detailed standard deviations in each bin in Fig. S12). While the standard error distribution of all bins for F/C and %m_F_ was the lowest (around ~ 7% ± approx. 7%), that for %n_F_ was medium (range of ~ 25%), and that for H/F was very large (up to 300%). This results from the fact that for highly fluorinated compounds, H/F becomes very small and close to zero (H = 1 or even 0 if F is very high) and in case of the standard error, the standard deviation is divided by this very small value of H/F (see also H/F plot in Fig. S13 close to the origin). Therefore, a prediction of H/F is not possible with reasonable precision. However, the F/C ratio of a compound can be estimated depending on its position in the MD/C-m/C plot with meaningful accuracy (see Fig. 6). Examples for F/C predictions from the mean matrix for the above PFAS examples are given in Table 1. With the estimated F/C ratio, the number of F atoms can further be calculated, since the number of C atoms is known. Furthermore, with the mass and number of F atoms, even %m_F_ can be estimated. For that purpose, an Excel sheet with the mean matrix (and standard deviation) of the F/C ratio and corresponding MD/C and m/C-bins is provided as Electronic Supplementary Material.

This general estimation approach, however, should be handled with care, because compounds in the MD/C-m/C plot can deviate from a CHF composition strongly making predictions more difficult. Also, measurement artifacts (e.g., errors in ^13^C isotope intensity due to detector saturation) should be considered in this approach. Overall, however, such a prediction provides useful information and a rough estimate of fluorine content of a measured feature based only on accurate mass measurements.

## Conclusions and implication for PFAS NTS

Overall, the MD/C-m/C approach is a highly promising tool for data reduction in NTS measurements and, therefore, to improve and accelerate non-targeted identification of highly fluorinated PFAS in highly complex samples. Since the prioritization does not require MS/MS data or several homologues, it is especially valuable in trace analysis approaches. It can efficiently be used to select potential PFAS candidates with high fluorine content for subsequent MS/MS experiments at a high probability. This increases the identification throughput since broad MS/MS coverage can be time consuming (multiple measurements per sample). Furthermore, during suspect screening approaches, the number of features can be substantially reduced to potential PFAS compounds so that the false positive rate (which is often very high, when using large PFAS lists) can be kept in a manageable extent. Special care should be taken when considering features at very high or low signal intensity, because of increased uncertainty of the determination of the ^13^C isotope abundance (and therefore MD/C and m/C). In the case of signals at or near MS detector saturation (e.g., in highly contaminated samples), the number of C atoms will be likely overestimated, shifting a feature to a lower m/C (stronger overlap with CH compounds) and lower MD/C. Therefore, it must be noted that both values are always subject to a certain error (e.g., ± 10%). However, if the MD/C of a feature is close to − 0.003 and m/C close to 50, there is a high probability that this indicates a highly fluorinated compound. Additionally, when the structure of a compound is known, the intrinsic property of the MD/C-m/C to cluster compounds of high elemental similarity can be used to get potential information on features that plot closely to known compounds.

In particular, we want to highlight the advantages of this approach over the use of MD only. Additionally, the MD/C-m/C position allows preliminary estimates on the elemental composition of a feature of interest.

We recommend an inside-out sequence of feature prioritization of HRMS data in an elliptical shape (starting from the CF_2_ location) along the CH_x_F_2-x_-line (see e.g., Eqs. 3–5, the PFAS region shown in Fig. 2c, or Figs. S9 and S10).

In addition, besides PFAS, the MD/C-m/C approach is highly promising for other compound classes like iodinated compounds (e.g., iodine-containing disinfection byproducts which are highly toxic [33]) since they are even better separated from non-halogenated features in both dimensions due to their high m/C and low MD/C from to the contribution of I. Overall, we hope that this approach will be adapted by a wide range of PFAS researchers as well as analytical laboratories.

## Supplementary Information

Below is the link to the electronic supplementary material.

Supplementary file1 (PDF 2710 KB)

Supplementary file2 (XLSX 88.9 KB)

## Abbreviations

F/C: Fluorine to carbon ratio (number)
H/C: Hydrogen to carbon ratio (number)
m/C: Mass to carbon value
MD/C: Mass defect to carbon value
NOM: Natural organic matter
OCs: Organic contaminants
PFAS: Per- and polyfluoroalkyl substances
%m_F_: Mass percentage of fluorine in a chemical formula
%n_F_: Molar percentage of fluorine in a chemical formula
