# Supplementary material for: Efficient PFAS prioritization in non-target HRMS data: systematic evaluation of the novel MD/C-m/C approach
Source: Anal Bioanal Chem. 2023 Feb 24;415(10):1791–801. doi: 10.1007/s00216-023-04601-1 (PMC10049945; doi:10.1007/s00216-023-04601-1)
Supplement: Supplementary file 1 — Supplementary file1 (PDF 2710 KB) [file 216_2023_4601_MOESM1_ESM.pdf]

<u>**Supporting Information**</u>

Efficient PFAS Prioritization in Non-Target HRMS Data: Systematic Evaluation of the novel MD/C-m/C Approach

Jonathan Zweigle,[+] Boris Bugsel,[+] Christian Zwiener[+,*]

[+]Environmental Analytical Chemistry, Department of Geosciences, University of Tübingen, Schnarrenbergstraße 94-96, 72076 Tübingen, Germany

[*]Corresponding author.

Contents

ABBREVIATIONS & VARIABLES 1
Supporting data........ 2
Fig. S 1: True mass defect (MD) vs calculable MD for PFAS, NOM [1], and organic contaminants (OCs). 2
Fig. S 2: Examples for PFAS with $\%m_F \geq 60\%$ that fall along the CH_xF_{2-x} and the CF_x-line. 2
Fig. S 3: Schematic explanation of matrix calculations. 3
Fig. S 4: Example for the calculation of the overlapping regions of PFAS with organic contaminants (OCs). 3
Fig. S 5: 2D histograms of the positions of PFAS, organic contaminants (OCs), and NOM compounds in the MD/C−m/C plot. 4
Fig. S 6: Further representation of the overlap of PFAS with organic contaminants (OCs). 5
Fig. S 7: Examples of four experimental MD/C-m/C plots from extracts of PFAS contaminated agricultural soils measured by Bugsel and Zwiener 2020 [2]. 5
Fig. S 8: Positions of 90% 95% and, 97% of the organic contaminants (OCs) with the true and the calculable MD (determined by rounding: MD = Exact mass – Integer mass)........ 6
Fig. S 9: Shifting and rotating the MD/C−m/C data. 6
Fig. S 10: Contour lines (black) of Equation 5 with $\lambda = 3000$ for PFAS prioritization in the MD/C−m/C plot........ 6
Fig. S 11: Dependency of the mean of the standard error matrix and the standard deviation of the standard error matrix of F/C, H/F, %mF, and $\%n_F$ on grid size........ 7
Fig. S 12: Standard deviation of F/C, H/F, $\%m_F$, and $\%n_F$ (shown as color bars) for PFAS with $\%m_F > 50\%$ in the MD/C−m/C plot 8
Fig. S 13: Predicted H/F, $\%m_F$, and $\%n_F$ based on the MD/C−m/C position in the respective mean matrix vs. real values for 52769 PFAS with $\%m_F > 50\%$. 8
REFERENCES 8

Abbreviations & Variables

F/C	Fluorine to carbon ratio (number)
H/C	Hydrogen to carbon ratio (number)
m/C	Mass to carbon value
MD/C	Mass defect to carbon value
NOM	Natural organic matter
OCs	Organic contaminants
PFAS	Per- and polyfluoroalkyl substances
$\%m_F$	Mass percentage of fluorine in a chemical formula
$\%n_F$	Molar percentage of fluorine atoms in a chemical formula

Supporting data

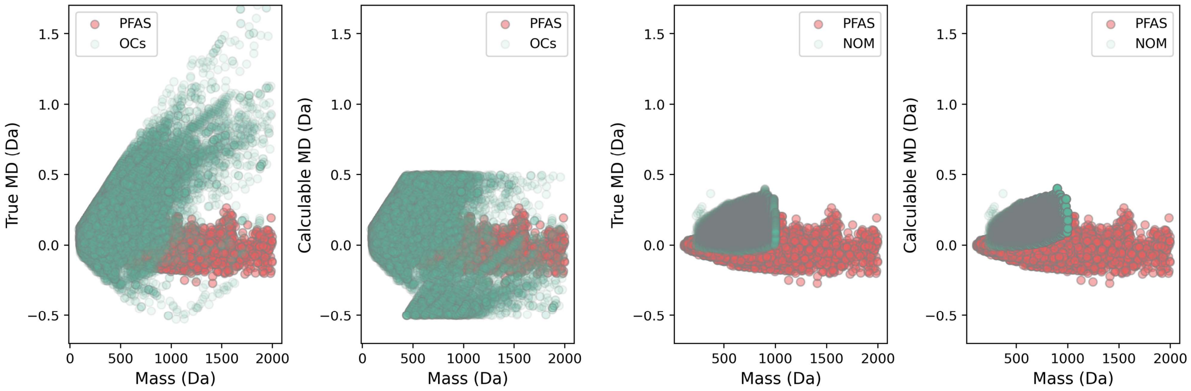

Fig. S 1: True mass defect (MD) vs calculable MD for PFAS, NOM [1], and organic contaminants (OCs). Obviously, numerous compounds exceed a mass defect of +0.5 Da, shifting them erroneously into the negative MD range which leads to overlapping with PFAS (left plot). This is not the case for NOM features.

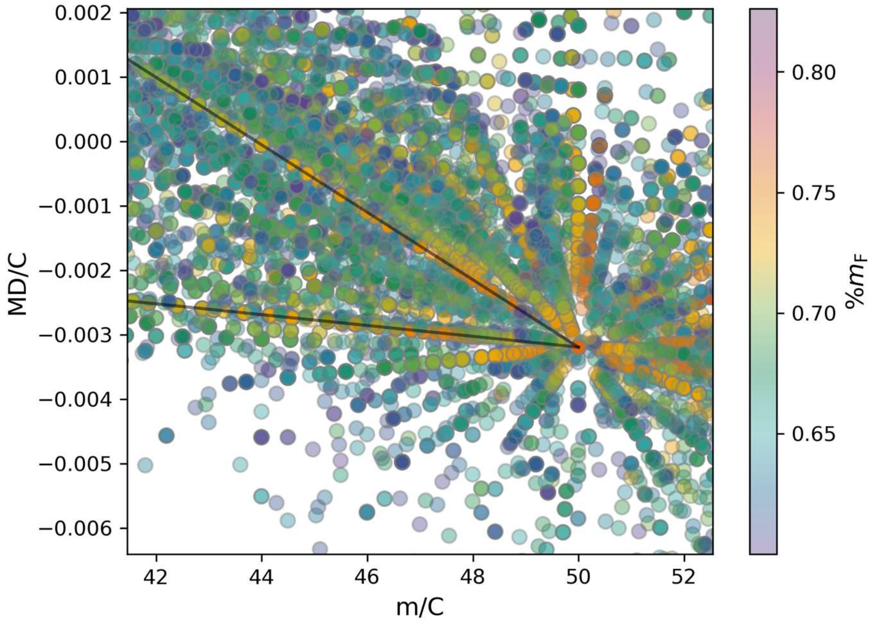

Fig. S 2: Examples for PFAS with %m_F ≥ 60% that fall along the CH_xF_{2-x} and the CF_x-line. The color bar corresponds to the respective %m_F. For details on the lines see Equation 1 and 2.

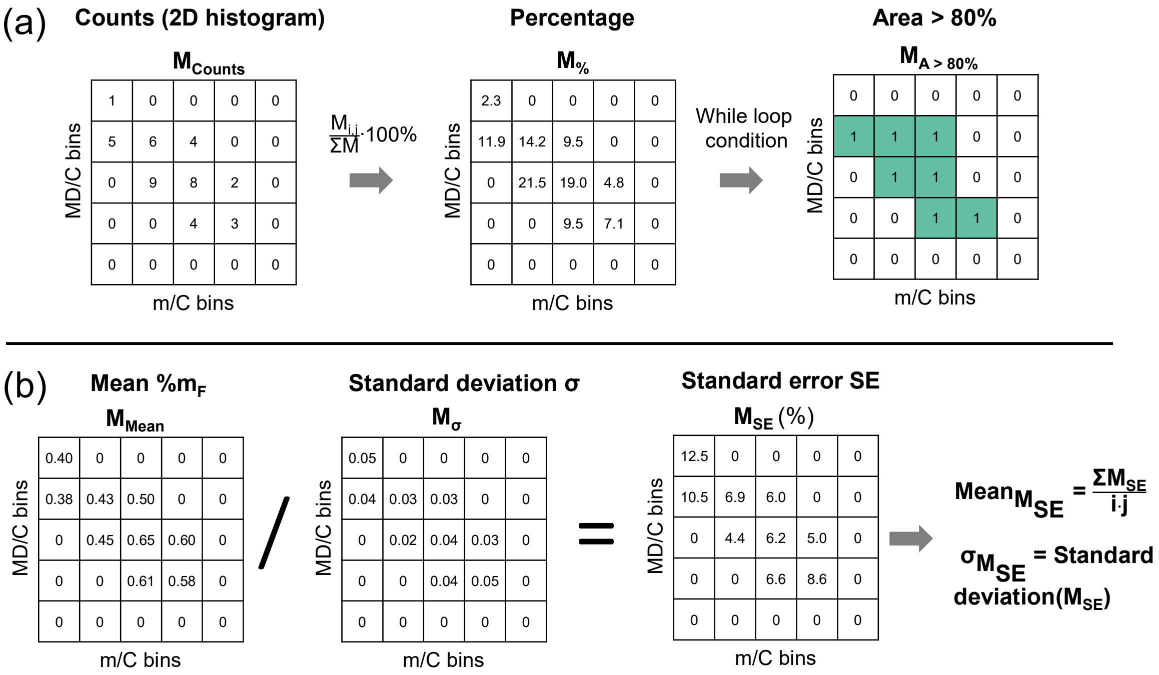

Fig. S 3: Schematic explanation of matrix calculations. (a) To find the area of a certain percentage of compounds in the MD/C−m/C plot the 2D histogram was normalized to its sum (percent compounds in each entry) and a while loop was used to find the area of x% in decreasing order (see also Fig. S4). (b) For F/C, H/F, $\%m_F$, and $\%n_F$ the mean and standard deviation matrices (see Fig. 5 and Fig. S12, for standard error distribution see Fig. 6) of PFAS with $\%m_F > 50\%$ that fall in each bin were calculated. The standard deviation matrix was divided by the mean matrix to obtain a matrix with the standard errors. For the grid size dependent standard error, the mean and standard deviation of the whole standard error matrix were plotted against grid size (Formulas for $Mean_{M_{SE}}$ and $\sigma_{M_{SE}}$, see also Fig. S11).

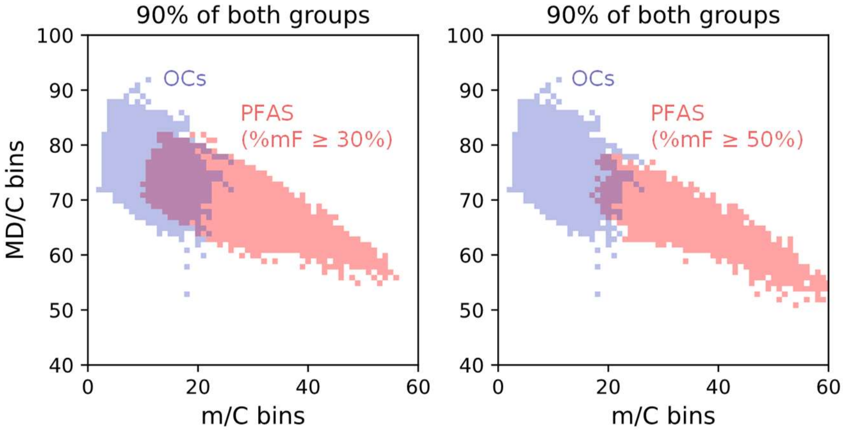

Fig. S 4: Example for the calculation of the overlapping regions of PFAS with organic contaminants (OCs). (Left) 90% of PFAS with $\%m_F > 30\%$ vs. 90% of OCs and (right) PFAS with $\%m_F > 50\%$ vs. 90% of OCs in a 100×100 binned grid. For continuous simulations see Fig. 5.

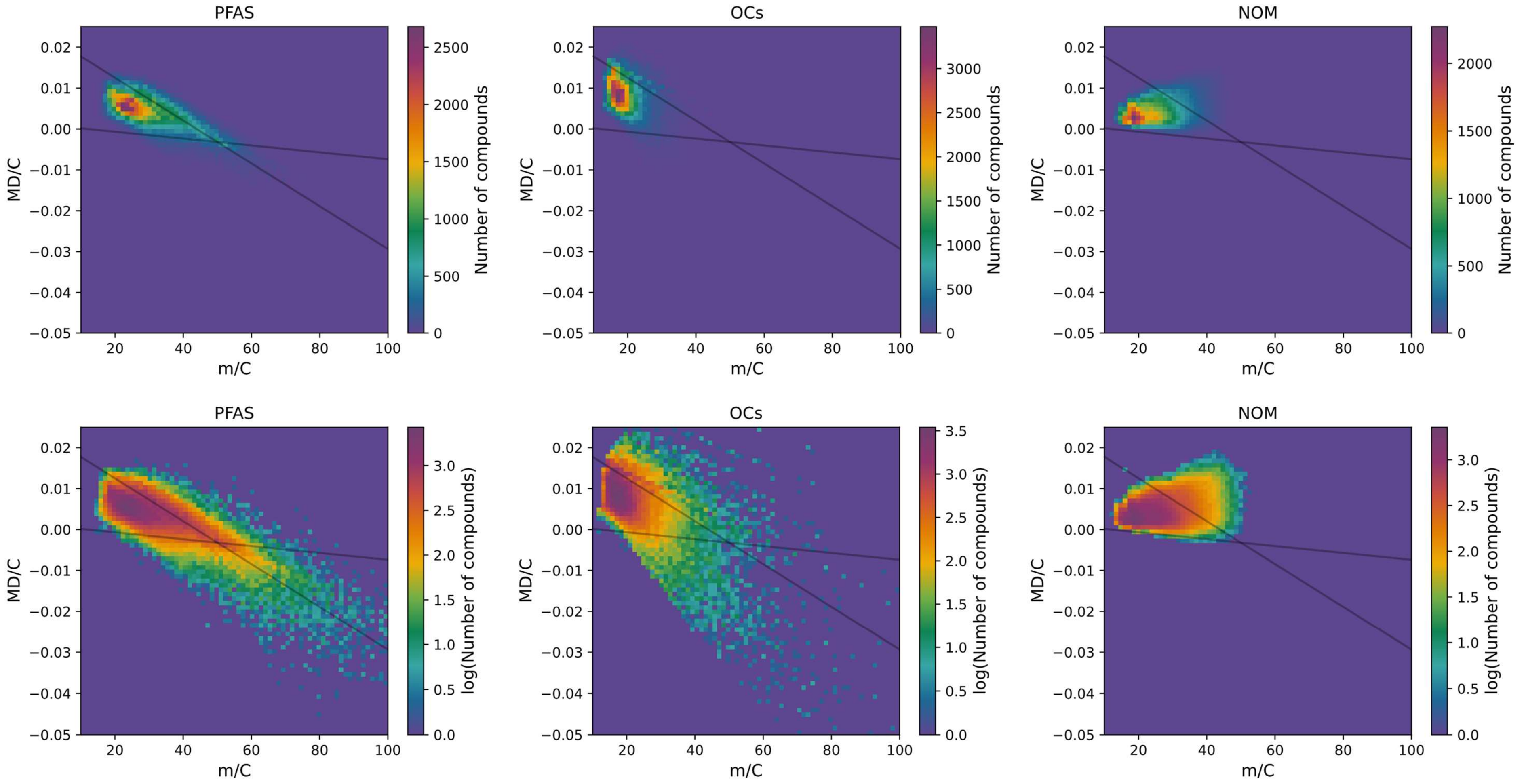

Fig. S 5: 2D histograms of the positions of PFAS, organic contaminants (OCs), and NOM compounds in the MD/C−m/C plot. Data is shown both in linear and log scale. In total, 210,091 PFAS, 159,236 OCs, and 124,782 NOM compounds are shown in a 70 × 70 bin grid.

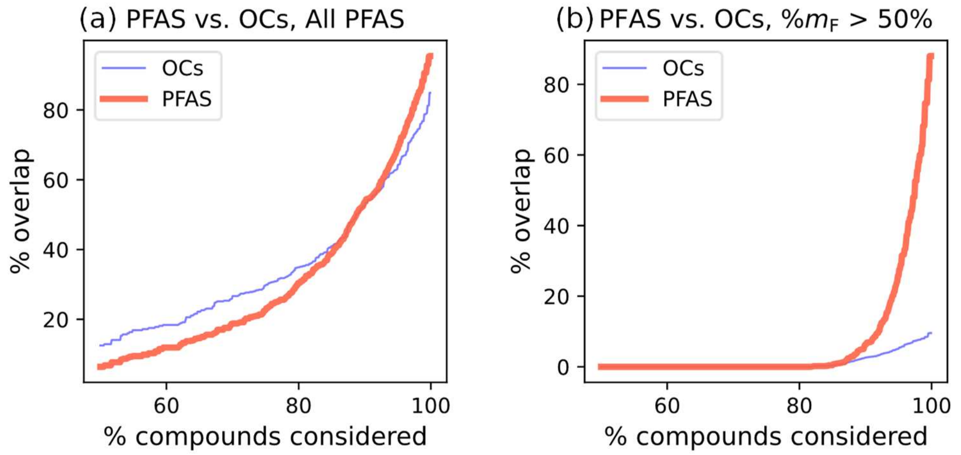

Fig. S 6: Further representation of the overlap of PFAS with organic contaminants (OCs). (a) % Overlap of PFAS and OCs vs. percent compounds considered for calculating this overlap. (b) Similar calculation but only PFAS with $\%m_F > 50\%$ were considered. The % compounds considered was calculated by summing up bins in decreasing order of compounds present.

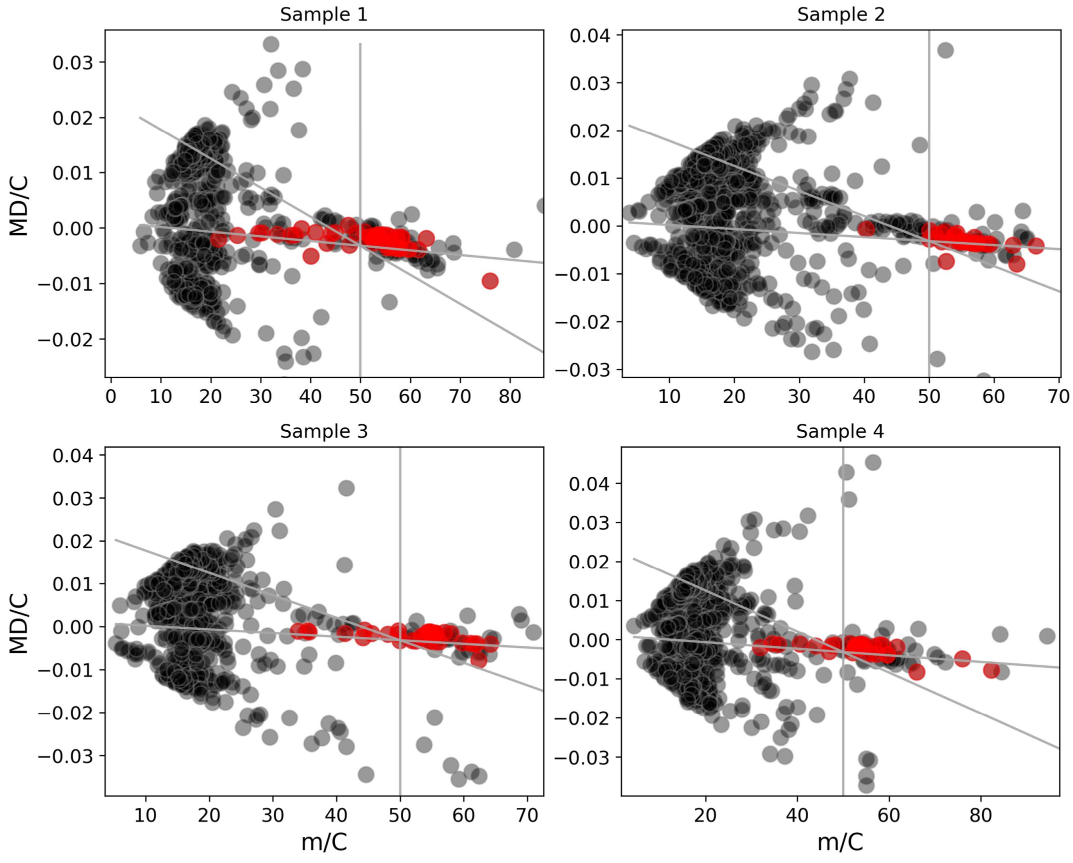

Fig. S 7: Examples of four experimental MD/C-m/C plots from extracts of PFAS contaminated agricultural soils measured by Bugsel and Zwiener 2020 [2]. Samples were measured by high-performance liquid chromatography quadrupole time-of-flight mass spectrometry and MS1 features above a certain intensity threshold are shown as black dots (details on the samples, sample preparation and identification can be found in [2]). Red dots are features that were identified as PFAS (e.g., perfluoroalkyl acids, polyfluoroalkyl phosphate esters, fluorotelomer carboxylic acids etc.). Overall an efficient separation from a significant fraction of matrix features can be observed which is generally concentrated along the proposed lines (see equation 1 and 2). The vertical line highlights m/C of 50 (= CF_2). Note: The sample numbers correspond to the sample numbers in [2].

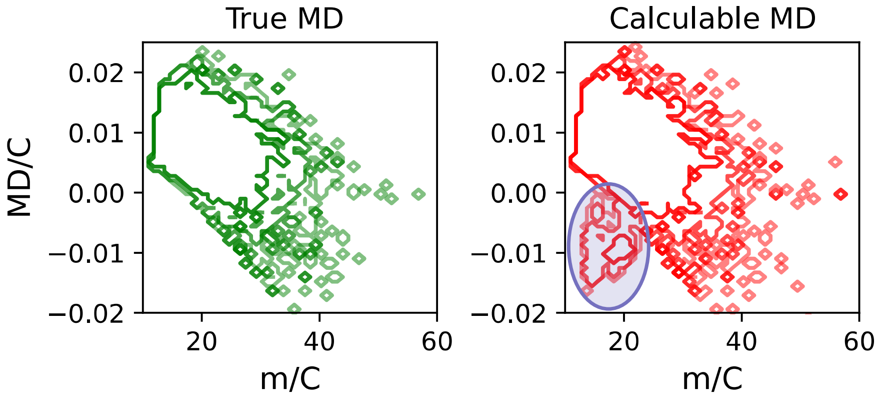

Fig. S 8: Positions of 90% 95% and, 97% of the organic contaminants (OCs) with the true and the calculable MD (determined by rounding: MD = Exact mass – Integer mass). It becomes obvious that the compounds with erroneously negative MD are unproblematic since they can be separated from PFAS in the m/C dimension. This is not the case when considering the MD alone (see Fig. S1).

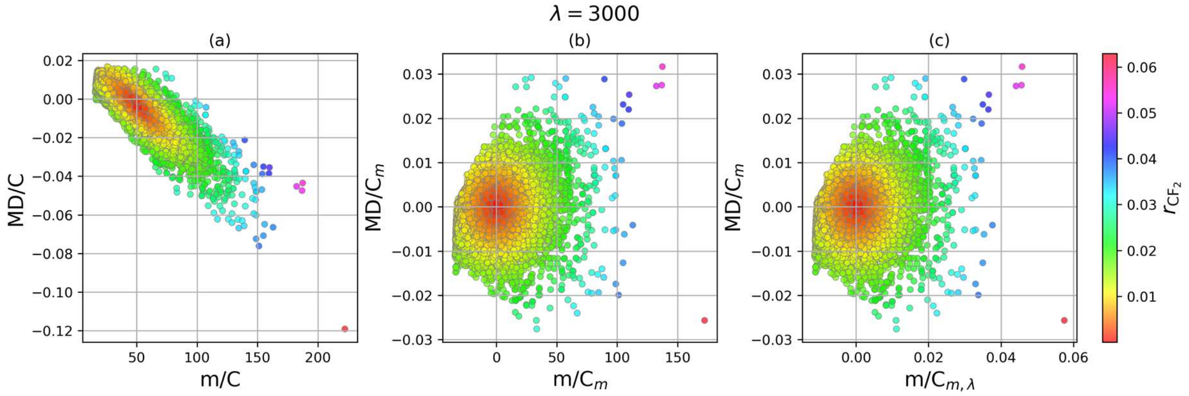

Fig. S 9: Shifting and rotating the MD/C−m/C data. (a) MD/C−m/C plot with calculated r_{CF_2} as color bar according to Equation 5 with λ = 3000 for PFAS with %m$_F$ > 20%. (b) Data shifted to the origin and rotated by the slope of the CH_xF_{2-x} line according to Equation 3 and 4. (c) Compression of m/C dimension through the factor λ.

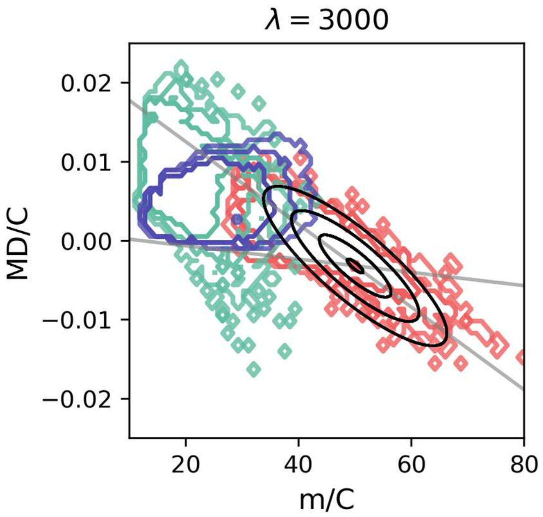

Fig. S 10: Contour lines (black) of Equation 5 with λ = 3000 for PFAS prioritization in the MD/C−m/C plot. Positions of OCs (green), NOM (blue) and PFAS with %mF > 55% (red) are shown. The colored contour lines delimit the positions of 80% (center), 90% (middle), and 95% of each group (see also Fig. 2).

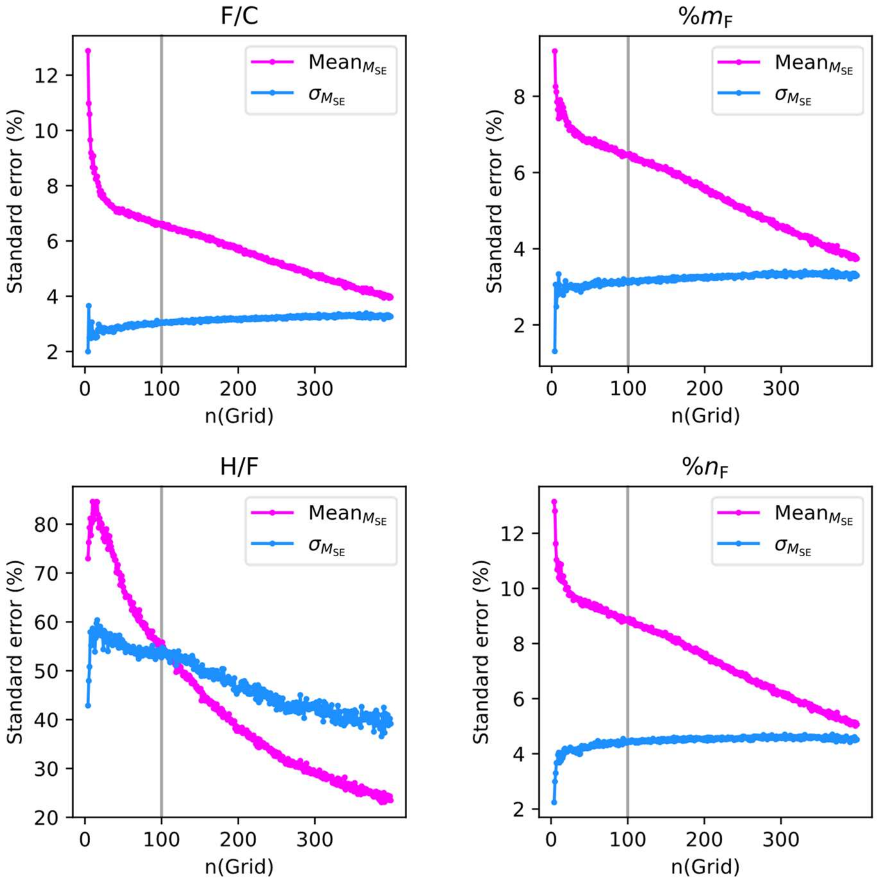

Fig. S 11: Dependency of the mean of the standard error matrix and the standard deviation of the standard error matrix of F/C, H/F, %mF, and %n_F on grid size (for schematic explanations see also Fig. S3b).

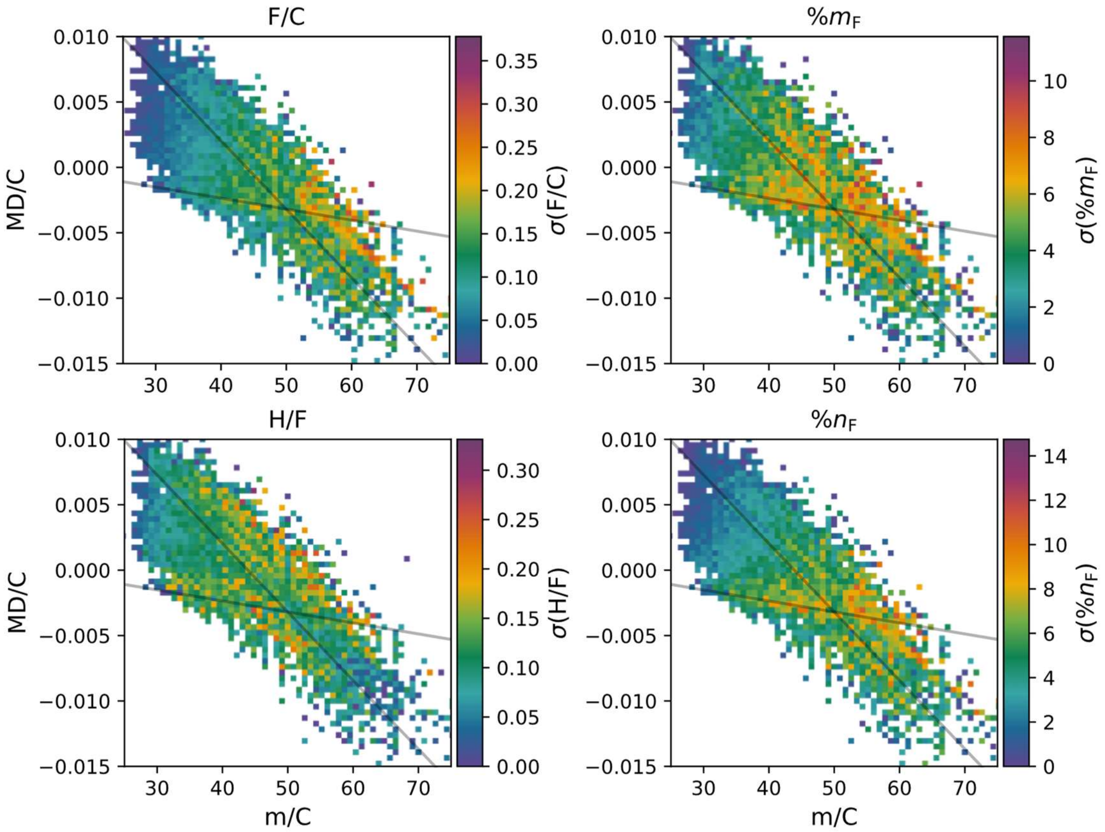

Fig. S 12: Standard deviation of F/C, H/F, %m_F, and %n_F (shown as color bars) for PFAS with %m_F > 50% in the MD/C−m/C plot (70 × 70 bins). For standard error distribution see Fig. 7. Note that the large standard error distribution of H/F result from the fact that in selected bins with highly fluorinated substances H/F approaches close to zero.

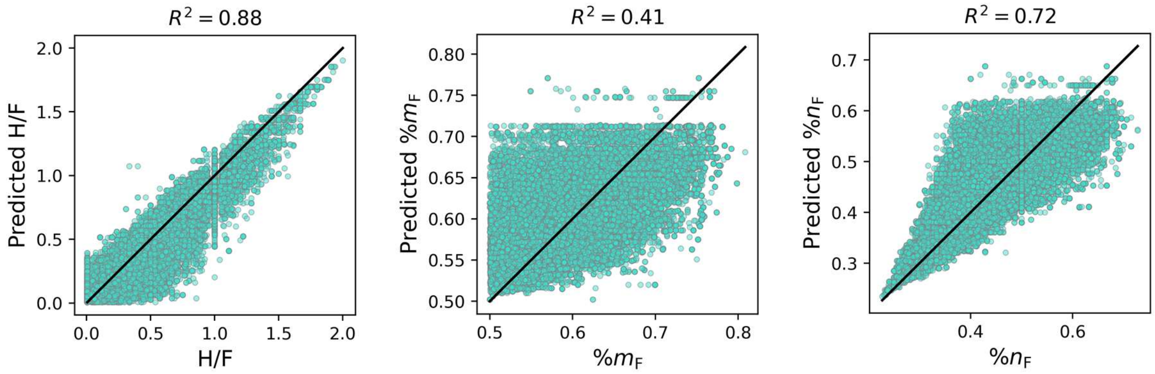

Fig. S 13: Predicted H/F, %m_F, and %n_F based on the MD/C−m/C position in the respective mean matrix vs. real values for 52769 PFAS with %m_F > 50%. Note that the large standard error of H/F (see Fig. 7) results from the variability in the low H/F range (compounds with much higher number of F than H), where is gets closer to zero.

References

1. Koch, B.P., et al., *Fundamentals of molecular formula assignment to ultrahigh resolution mass data of natural organic matter.* Anal Chem, 2007. **79**(4): p. 1758-63.
2. Bugsel, B. and C. Zwiener, *LC-MS screening of poly- and perfluoroalkyl substances in contaminated soil by Kendrick mass analysis.* Anal Bioanal Chem, 2020. **412**(20): p. 4797-4805.
